# Bicruciate-retaining versus cruciate-retaining total knee arthroplasty in implant fixation: a 10-year follow-up of a randomized RSA trial

**DOI:** 10.2340/17453674.2026.46805

**Published:** 2026-09-17

**Authors:** Tomofumi KINOSHITA, Kristian R L MORTENSEN, Lina H INGELSRUD, Omar MUHAREMOVIC, Kirill GROMOV, Anders TROELSEN

**Affiliations:** 1 Department of Orthopaedic Surgery, Copenhagen University Hospital Hvidovre, Copenhagen, Denmark; 2 Department of Clinical Medicine, Copenhagen University, Copenhagen, Denmark; 3 Department of Radiology and Nuclear Medicine, Copenhagen University Hospital Hvidovre, Copenhagen, Denmark; 4 Department of Orthopaedic Surgery, Ehime University Graduate School of Medicine, Ehime, Japan

## Abstract

**Background and purpose:**

Bicruciate-retaining (BCR) total knee arthroplasty (TKA) aims to restore physiological knee kinematics and joint stability by preserving both cruciate ligaments, potentially improving patient satisfaction. However, long-term clinical outcomes and tibial component migration remain insufficiently understood. We therefore aimed to evaluate long-term implant fixation and clinical outcome after BCR-TKA compared with cruciate-retaining (CR) TKA.

**Methods:**

We performed a long-term follow-up of a randomized controlled trial using radiostereometric analysis (RSA) to compare tibial implant migration between BCR-TKA and CR-TKA. The primary outcome was maximum total point motion (MTPM) of the tibial component, measured with model-based RSA at 3 months, 1, 2, 5, and 10 years. Secondary outcomes included the Oxford Knee Score (OKS) and Forgotten Joint Score (FJS) at identical time points. Mortality, reoperations, and revisions were recorded.

**Results:**

25 patients were included in each group. At 10 years, the estimated mean MTPM was 0.88 mm (95% confidence interval [CI] 0.64–1.12) in the BCR group and 0.64 mm (CI 0.40–0.88) in the CR group (between-group difference −0.23 mm, CI −0.57 to 0.10). Estimated changes in OKS and FJS showed no evidence of differing longitudinal trajectories between groups. 2 revisions occurred in the BCR group, whereas none occurred in the CR group.

**Conclusion:**

No significant differences were detected between BCR-TKA and CR-TKA in tibial implant migration or patient-reported outcomes over the 10-year follow-up. In contrast, the incidence of complications was higher in the BCR group, indicating that careful patient selection and optimization of surgical strategies remain important for BCR-TKA.

Despite the proven clinical effectiveness of total knee arthroplasty (TKA), reproducing native knee kinematics remains challenging. Differences between native and postoperative knee motion have been consistently reported [1] and are thought to contribute to suboptimal patient satisfaction [2]. Furthermore, numerous studies have highlighted the occurrence of non-physiological kinematics, such as paradoxical anterior movement [3], and have pointed out the influence of anterior cruciate ligament (ACL) resection in conventional TKA.

To address these issues, bicruciate-retaining (BCR) TKA has gained attention. BCR-TKA is designed to preserve both the posterior cruciate ligament and the ACL, with the goal of restoring the kinematics and stability of a normal knee. However, the complexity of the surgical technique and challenges in achieving proper ligament balance have led to concerns regarding postoperative clinical results [4]. Additionally, compared with conventional TKA, BCR-TKA has been reported to be associated with a higher revision rate, which also remains a significant concern [5].

Long-term prospective studies are essential for evaluating differences in clinical outcomes between various implant types. However, randomized controlled trials (RCTs) comparing BCR-TKA and conventional TKA remain limited.

Beyond patient-reported outcomes, implant fixation and stability represent another critical aspect of long-term TKA performance. Early postoperative migration of prosthetic components has been reported to predict the risk of future revision surgery [6]. From this perspective, detailed evaluation of implant status in the early postoperative period is critically important. Radiostereometric analysis (RSA) has emerged as a technique that enables highly accurate assessment of implant migration from the early postoperative phase [7]. However, no studies have reported long-term (≥ 10-year) migration of tibial implants using RSA in BCR-TKA compared with conventional TKA.

Our study reports the 5- and 10-year outcomes of a RCT comparing BCR-TKA and cruciate-retaining (CR) TKA. The aim was to (i) evaluate the difference in tibial implant migration between the 2 groups 10 years after surgery, (ii) compare the difference in patient-reported outcome measures (PROMs) between the 2 groups, and (iii) evaluate the frequency of all-cause mortality, reoperation, and revision surgeries in the 2 groups of TKA. We hypothesized that tibial implant migration at 10 years would not differ between BCR-TKA and CR-TKA.

## Methods

### Study design

This study is a prespecified 10-year follow-up of a previously published single-blind randomized controlled trial registered at ClinicalTrials.gov (NCT01966848) [8]. The trial was conducted and reported according to CONSORT and RSA-specific recommendations [9,10]. Randomization used a computer-generated sequence with allocation concealed in sequentially numbered, sealed opaque envelopes. Patients remained blinded until the 2-year follow-up, at which point they were informed of the implant design received. The original sample size was based on detecting a 0.2-mm difference in mean component migration (SD 0.2) with 80% power at α = 0.05, requiring 18 participants per group; we therefore planned to include 25 per group to allow for attrition [11].

### Eligibility and recruitment

Between January 20, 2014, and September 8, 2015, patients meeting the eligibility criteria were enrolled and randomized at a high-volume orthopedic department in Denmark. Eligible participants were adults (≥ 18 years) with knee osteoarthritis scheduled for primary unilateral cemented total knee arthroplasty. Additional inclusion criteria comprised sufficient proficiency in Danish, the ability to provide written informed consent, and an expected ability to comply with postoperative follow-up visits and completion of PROMs.

Patients were excluded if they had severe systemic comorbidity (American Society of Anesthesiologists classification [ASA] > 3), terminal illness, inflammatory arthropathies such as rheumatoid arthritis, or pharmacologically treated osteoporosis. Further exclusion criteria included medical conditions known to influence pain perception (e.g., diabetic neuropathy), a history of open or arthroscopic cruciate ligament surgery of the index knee, previous high-energy knee trauma or cruciate ligament rupture, or clinical signs suggestive of cruciate ligament insufficiency. Screening for eligibility and all surgical procedures were performed by 2 experienced orthopedic surgeons using a standardized protocol. Baseline demographic and clinical characteristics were comparable between the 2 treatment groups.

During follow-up, losses occurred due to death, revision surgery, and refusal of radiographic assessment. Reasons for exclusion from the RSA analysis included revision surgery, death, refusal of radiographic assessment, insufficient visibility of tantalum beads, or absence of the 10-year radiographic examination.

### Surgical techniques

Patients were randomly assigned to undergo either BCR-TKA (Vanguard XP, Zimmer Biomet, Warsaw, IN, USA) or CR-TKA (Vanguard CR, Zimmer Biomet). All procedures were performed by the same 2 senior orthopedic surgeons using a standardized operative protocol. Prior to study initiation, both surgeons completed dedicated training with the BCR implant to ensure adequate technical familiarity. Baseline demographic characteristics and radiographic severity of osteoarthritis were comparable between groups (see Table 1).

**Table 1:** Preoperative patient characteristics. Values are mean (SD) unless otherwise stated

Preoperative characteristic	BCR (n = 25)	CR (n = 25)
Age	66 (9.2)	70 (6.8)
Female sex, n	18	14
Body mass index	29 (5.5)	29 (5.0)
ASA class, n		
1	12	7
2	13	15
3	–	3
Kellgren–Lawrence, n		
2	4	1
3	9	10
4	12	14
Oxford Knee Score	21 (6.6)	24 (6.6)
Forgotten Joint Score-12	16 (16)	21 (15)

BCR: bicruciate-retaining; CR: cruciate-retaining; SD: standard deviation; ASA: American Society of Anesthesiologists.

All surgeries were performed through a standard medial parapatellar approach. Bone preparation followed a measured resection technique according to the manufacturer’s guidelines, and patellar resurfacing was routinely performed. Femoral alignment was established using an intramedullary guide, with component rotation determined by standard anatomic landmarks. Tibial alignment was achieved using an extramedullary guide, with posterior tibial slope adjusted to the individual anatomy within a maximum of 7°.

In BCR-TKA, the central tibial bone island was preserved, whereas in CR-TKA, the posterior cruciate ligament insertion was carefully maintained. Soft-tissue balance and knee stability were assessed intraoperatively throughout the range of motion. All components were cemented using a surface cementation technique with Optipac Refobacin R bone cement (Zimmer Biomet). A tourniquet was not used. Postoperative rehabilitation protocols were identical between groups, allowing immediate full weightbearing as tolerated.

### Outcome measures

#### RSA analyses (primary outcomes)

The prespecified primary outcome in the original study was migration of the tibial component assessed at 2 years postoperatively using model-based RSA [4]. The primary outcome of the current study was tibial component maximum total point motion (MTPM), which represents the largest 3-dimensional translation of any point on the tibial component relative to the surrounding bone [10]. RSA enables high-precision quantification of implant motion by measuring translations (in millimeters) along, and rotations (in degrees) about, the 3 orthogonal axes. The RSA setup used in the current study has been described previously [8]. RSA was conducted according to a standardized imaging protocol. 2 ceiling-mounted radiographic tubes were positioned symmetrically, each angled 23° from the vertical plane, producing a total angulation of 46° across the joint of interest. The source-to-detector distance was maintained at 150 cm. A uniplanar calibration cage (CarbonBox021Kopenhagen; MEDIS Medical Imaging Systems BV, Leiden, The Netherlands) was placed between the participant and the detectors to facilitate 3-dimensional model reconstruction. Image acquisition was performed using a digital radiography system with a Carestream DRX-1 detector (Carestream Health, Rochester, NY, USA). All examinations were obtained with the knee in an unloaded condition; patients were placed in the supine position with the operated-on leg oriented parallel to the y-axis of the calibration cage. Implant migration was assessed by measuring translations and rotations along 3 perpendicular axes: the x-axis (medial–lateral), y-axis (proximal–distal), and z-axis (anterior–posterior) after normalizing all knees to right-sided knees.

RSA examinations were performed immediately after surgery to establish the reference position of the implant in the bone, with subsequent follow-up assessments conducted at 3 months, 1 year, 2 years, 5 years, and 10 years. All RSA acquisitions and analyses were carried out according to a standardized protocol with predefined quality criteria applied consistently throughout the study [10]. All RSA measurements were performed by 2 trained observers using dedicated model-based RSA software (version 4.11; RSA*core*, Leiden, the Netherlands). During surgery, 6–8 1.0-mm tantalum markers (RSA Biomedical, Umeå, Sweden) were implanted in the proximal tibial metaphysis to enable accurate tracking of component motion. RSA examinations were excluded from migration analyses if fewer than 3 markers were identifiable around the tibial component. Measurement precision of the RSA setup was evaluated at the 3-month follow-up by acquiring duplicate stereoradiographs for each participant. RSA repeatability was calculated as 1.96 × √2 × SD of the paired differences, and was 0.26 mm with the current RSA setup [8].

#### Secondary outcomes

Patient-reported outcomes included the Oxford Knee Score (OKS) and the Forgotten Joint Score-12 (FJS). The OKS ranges from 0 to 48 and the FJS from 0 to 100, with higher scores indicating better outcomes for both measures. The reported MCIDs are 5 points for the OKS and 12.5 points for the FJS [12,13]. Both instruments were collected preoperatively and 3 months, 1, 2, 5, and 10 years after surgery. Changes in OKS and FJS from baseline were calculated for each follow-up time point and used for between-group comparisons.

### Statistics

Longitudinal between-group comparisons were performed using a likelihood-based mixed model for repeated measures (MMRM) including allocated group, follow-up, and group-by-follow-up interaction as fixed effects. This approach was used for primary outcome (tibia MTPM) and for secondary PROM-change outcomes. All models used all observations recorded under the assumption that missing data was missing at random. Model assumptions were inspected using Q–Q plots, and were deemed to be valid. The significance of the fixed effects and the group-by-time interaction was assessed using analysis of variance. Estimated means (least-squares means) with 95% confidence interval (CI) were calculated for each measurement, group, and follow-up, and between-group differences were calculated as mean and 95% CI. Accordingly, the study was not specifically powered to detect clinically meaningful differences in secondary outcomes, including patient-reported outcome measures. All statistical analyses were conducted using R software (RStudio 2024.04.2; R Foundation for Statistical Computing, Vienna, Austria). A 2-sided P value < 0.05 was considered statistically significant.

### Ethics, data sharing plan, funding, use of AI, and disclosures

Ethical approval (H-1-2013-086) and data processing authorization (HVH-2013-050) were obtained. The data supporting the findings of this study is available from the corresponding author upon reasonable request. No funding was received for this study. ChatGPT (OpenAI) was used solely to improve the readability and language of the manuscript. The authors reviewed and approved all changes and take full responsibility for the content of the manuscript. Complete disclosure of interest forms according to ICMJE are available on the article page, doi: 10.2340/17453674.2026.46805

## Results

**Figure 1:**
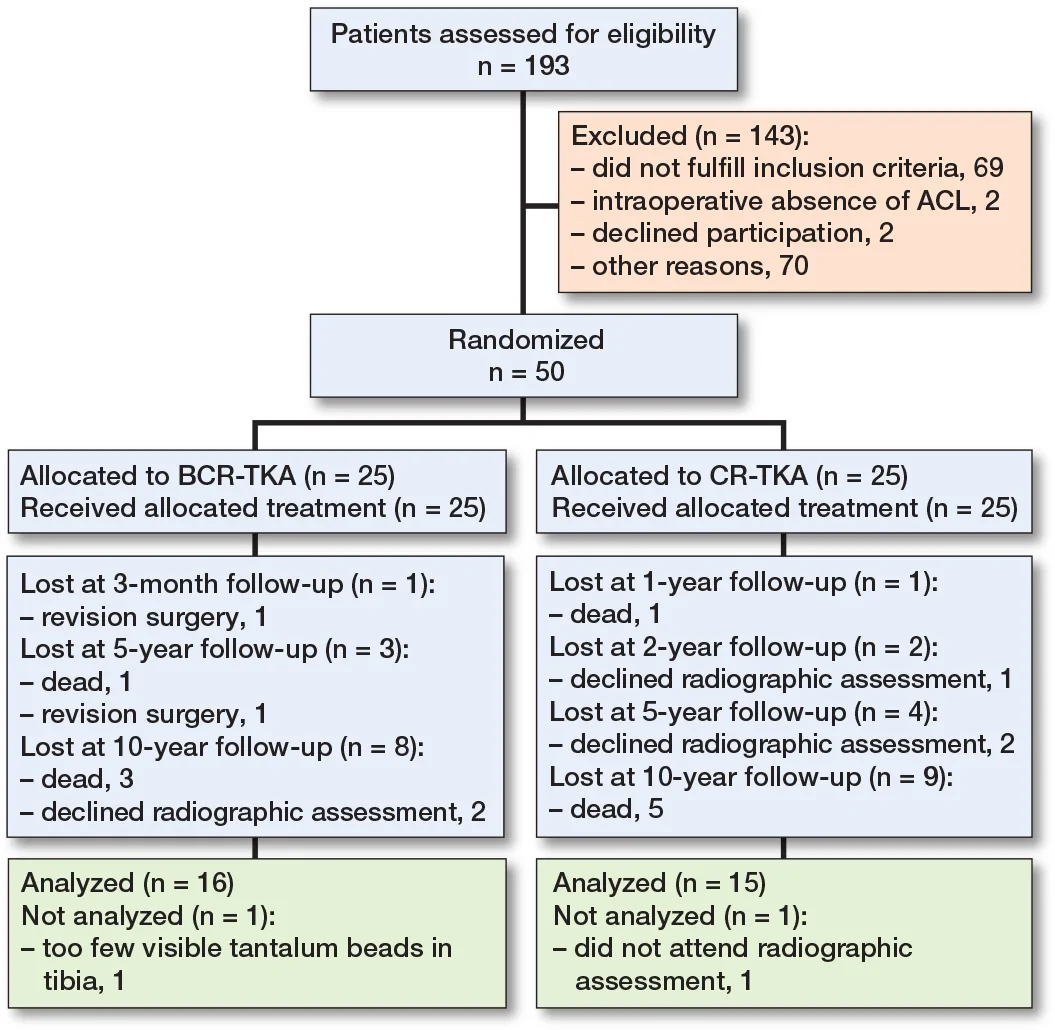
Flow diagram illustrating patient enrollment and follow-up. ACL: anterior cruciate ligament, BCR: bicruciate-retaining, CR: cruciate-retaining, TKA: total knee arthroplasty,

50 patients were randomized (25 BCR-TKA and 25 CR-TKA). At the 10-year follow-up, 16 patients in the BCR-TKA group and 15 patients in the CR-TKA group remained available for RSA analysis after losses due to revision, death, withdrawal, or missing radiographic assessment (Table 1, Figure 1).

### Tibial implant migration

**Figure 2:**
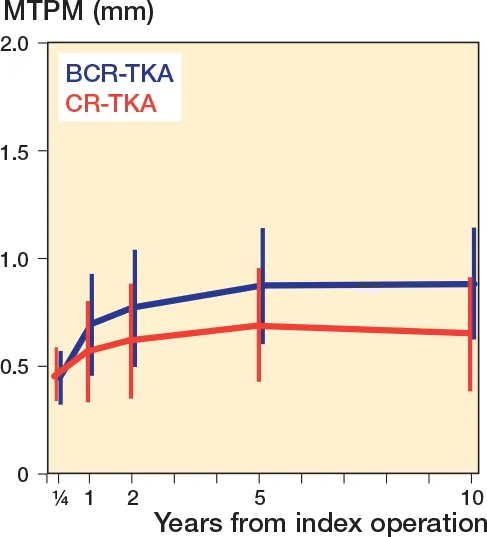
Mean tibial maximum total point motion (MTPM) in the BCR and CR groups derived from the mixed model for repeated measures (MMRM) at 3 months, 1 year, 2 years, 5 years, and 10 years postoperatively in the BCR (blue line) and CR (red line) groups. Error bars indicate 95% confidence intervals (CIs). BCR: bicruciate-retaining; CR: cruciate-retaining; TKA: total knee arthroplasty.

MTPM increased over time in both groups (Figure 2). At 10 years, the estimated mean MTPM was 0.64 mm (CI 0.40–0.88) in the CR group and 0.88 mm (CI 0.64–1.12) in the BCR group, corresponding to an estimated between-group difference of –0.23 mm (CI –0.57 to 0.10). The longitudinal trajectories did not differ between groups (group × time interaction, P = 0.5), and no significant between-group differences were observed at any follow-up (Table 2).

**Table 2:** Mixed model for repeated measures-estimated mean MTPM (mm) at each follow-up with 95% confidence interval (CI)

Follow-up	BCR mean (CI)	CR mean (CI)	Group difference mean (CI)	P value
3 months	0.44 (0.33–0.54)	0.45 (0.35–0.56)	0.01 (–0.13 to 0.16)	0.8
1 year	0.68 (0.47–0.90)	0.56 (0.34–0.77)	–0.12 (–0.43 to 0.18)	0.4
2 years	0.76 (0.51–1.01)	0.61 (0.36–0.86)	–0.15 (–0.50 to 0.19)	0.4
5 years	0.87 (0.62–1.11)	0.68 (0.44–0.93)	–0.18 (–0.52 to 0.16)	0.3
10 years	0.88 (0.64–1.12)	0.64 (0.40–0.88)	–0.23 (–0.57 to 0.10)	0.2

MTPM: maximum total point motion; BCR: bicruciate-retaining; CR: cruciate-retaining.

Estimated mean tibial MTPM at each postoperative follow-up derived from the mixed model for repeated measures (MMRM). Values are estimated means with 95% confidence intervals. P values were obtained from the mixed model for repeated measures (MMRM) comparing the estimated trajectories between groups over the follow-up period.

Migration outliers were identified in 2 patients in the BCR group and 1 patient in the CR group, defined as an MTPM at 2 years exceeding the group-specific mean plus 2 standard deviations. In these patients, the majority of migration had occurred during the first postoperative year, with subsequent stabilization observed between 1 and 2 years (BCR: 2.8–3.0 mm and 1.8–2.3 mm; CR: 1.6–1.8 mm MTPM) [8]. Despite increased migration, all 3 patients demonstrated favorable clinical outcomes at their respective last clinical assessments conducted at either 5 or 10 years after surgery, with OKS values of 36, 47, and 48. No complication or revision occurred in the migration outlier group.

### Patient-reported outcome measure

**Figure 3:**
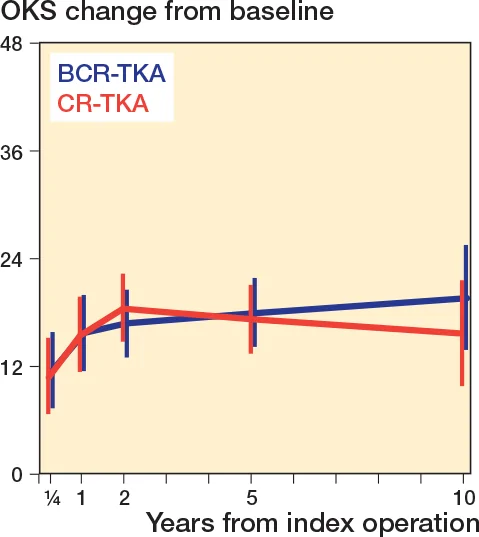
Mean change in Oxford Knee Score (OKS) from baseline derived from the MMRM at 3 months, 1 year, 2 years, 5 years, and 10 years postoperatively in the BCR (blue line) and CR (red line) groups. Error bars indicate CIs. For abbreviations, see Figure 2.

**Figure 4:**
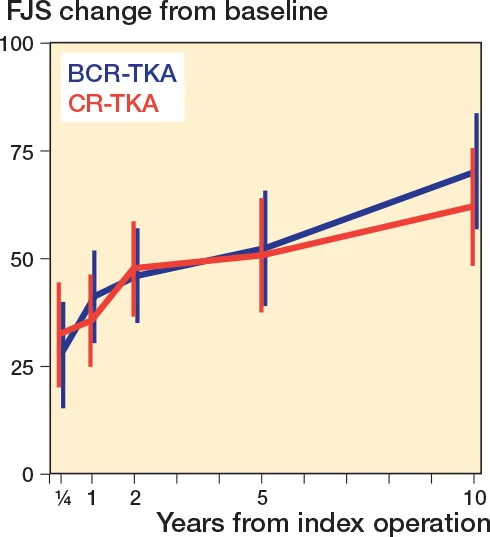
Mean change in Forgotten Joint Score-12 (FJS) from baseline derived from the MMRM at 3 months, 1 year, 2 years, 5 years, and 10 years postoperatively in the BCR (blue line) and CR (red line) groups. Error bars indicate CIs. For abbreviations, see Figure 2.

Change from baseline in OKS continued to improve until 2 years postoperatively and then remained stable throughout the remainder of the follow-up period (Figure 3). At 10 years’ follow-up, change from baseline OKS was 19.7 (CI 14.2–25.2) in the BCR group and 15.7 (CI 10.2–21.3) in the CR group. Between-group difference was –4.0 (CI –11.8 to 3.9). In change from baseline FJS, both groups showed signs of continuous improvement throughout the study period (Figure 4). At 10 years’ follow-up, change from baseline FJS was 70.1 (CI 57.4–82.9) in the BCR group and 62.0 (CI 49.0–74.9) in the CR group. Longitudinal trajectories of the change from baseline PROM (OKS, FJS) were not significantly different (group × time interaction, P = 0.2 and P = 0.1) for OKS and FJS respectively. There were no significant differences between the groups in any PROM at any follow-up (Tables 3 and 4).

**Table 3:** Mixed model for repeated measures-estimated mean OKS at each follow-up with 95% confidence interval (CI)

Follow-up	BCR mean (CI)	CR mean (CI)	Group difference mean (CI)	P value
3 months	12 (7.7–16)	11 (7.0–15)	–0.7 (–6.2 to 4.9)	0.8
1 year	16 (12–20)	16 (12–19)	–0.1 (–5.6 to 5.4)	>0.9
2 years	17 (13–20)	19 (15–22)	1.8 (–3.1 to 6.6)	0.5
5 years	18 (15–22)	17 (14–21)	–0.7 (–5.7 to 4.2)	0.8
10 years	20 (14–25)	16 (10–21)	–4.0 (–12 to 3.9)	0.3

OKS: Oxford Knee Score, BCR: bicruciate-retaining; CR: cruciate-retaining.

Estimated mean changes in OKS from baseline at each postoperative follow-up and P value was derived as described in Table 2.

**Table 4:** Mixed model for repeated measures-estimated mean FJS at each follow-up with 95% confidence interval (CI)

Follow-up	BCR mean (CI)	CR mean (CI)	Group difference mean (CI)	P value
3 months	28 (16–39)	32 (21–44)	4.7 (–12 to 21)	0.6
1 year	41 (31–51)	36 (26–45)	–5.6 (–20 to 8.4)	0.4
2 years	46 (36–56)	48 (37–58)	1.9 (–13 to 16)	0.8
5 years	52 (40–65)	51 (38–63)	–1.6 (–19 to 16)	0.9
10 years	70 (57–83)	62 (49–75)	–8.1 (–26 to 10)	0.4

FJS: Forgotten Joint Score-12; BCR: bicruciate-retaining; CR: cruciate-retaining.

Estimated mean changes in FJS from baseline at each postoperative follow-up and P value was derived as described in Table 2.

### Reoperations and revisions

No reoperations occurred in the CR group during the whole period. Within the first 5 years postoperatively, there were 3 cases of manipulation under anesthesia, 1 case of an intraoperative tibial plateau fracture that required revision at 6 months, and 1 case in which arthroscopic synovectomy was performed, followed by revision surgery. All of these events occurred in the BCR group. No new complications or reoperations were observed during the 5–10-year follow-up period.

## Discussion

We aimed to evaluate long-term implant fixation and clinical outcome after BCR-TKA compared with CR-TKA. We found that estimated between-group differences in tibial implant migration and patient-reported outcomes were small throughout the 10-year follow-up. However, the confidence intervals indicate that some uncertainty remains around the estimates for both implant migration and PROMs. In addition, the incidence of complications within the first 5 postoperative years was higher in the BCR group.

BCR-TKA was introduced as an alternative to CR-TKA to enhance joint stability through preservation of both cruciate ligaments [14]. Although early outcomes have been favorable, concerns regarding complications and revision rates have highlighted the need for long-term comparative evidence [15]. Long-term randomized data integrating RSA and patient-reported outcomes remains limited; therefore, this RCT provides a 10-year comparison of BCR-TKA and CR-TKA, enabling a comprehensive assessment of implant fixation and clinical performance. Mills et al. reported greater implant migration between 6 months and 1 year postoperatively in BCR-TKA than in CR-TKA [16]. In contrast, the present study found no significant differences in MTPM over the 10-year follow-up. Migration exceeding the revision risk threshold proposed by Pijls et al. (1-year MTPM 0.45–1.6 mm) [7] was observed in 2 BCR cases and 1 CR case; however, migration subsequently stabilized without substantial progression, and no revisions were required during follow-up.

Concerning PROMs assessing pain, function, and knee awareness, several randomized controlled trials and comparative studies have evaluated different implant designs in total knee arthroplasty. Lavoie et al. reported no significant differences in PROMs between posterior-stabilized (PS)-TKA and BCR-TKA at a mean follow-up of 39 months, although slightly greater knee flexion was observed in the PS-TKA group [17]. Similarly, Kyriakidis et al. found no significant differences in PROMs between BCR-TKA and PS-TKA at 2 years of follow-up [18]. In addition, Biazzo et al. reported that BCR-TKA demonstrated functional recovery comparable to that of CR-TKA in the short term [19]. Overall, previous studies have not demonstrated consistent superiority of BCR-TKA over other implant designs with respect to PROMs in accordance with our study. As also indicated by the present findings, concerns regarding complications and revision rates, which have reached 11–12% in some studies, remain major factors limiting the widespread adoption of BCR-TKA [5,20].

### Limitations

The relatively small sample size and the wide confidence intervals warrant caution when interpreting the findings. The reduced sample size is partly attributable to the long-term (10-year) follow-up design and patient dropouts, which may have limited the statistical power of both the PROM and RSA analyses at 10 years. Additionally, only tibial component migration was assessed using RSA, and the study was conducted at a single institution. Nevertheless, this randomized trial provides rare long-term data integrating RSA and patient-reported outcomes for the evaluation of BCR-TKA.

### Conclusion

No significant differences were detected between BCR-TKA and CR-TKA in tibial implant migration or patient-reported outcomes over the 10-year follow-up. In contrast, the incidence of complications was higher in the BCR group.

## Notes

TK: Formal analysis, investigation, writing – original draft, writing – review & editing; KRL: Methodology, investigation, formal analysis, writing – review & editing. OM: Investigation, writing – review & editing; LHI, KG: Methodology, writing – review & editing; AT: Conceptualization, methodology, writing – review & editing.

Handling co-editors: Li Felländer-Tsai and Robin Christensen

*Acta* thanks Harald Brismar and Sebastiaan van de Groes for help with peer review of this manuscript.
